# RNA duplex formation and competing endogenous RNA, proposed as mechanisms in regulating expression of natural antisense transcripts- from hypotheses to potential therapeutic applications

**DOI:** 10.3389/fmolb.2026.1800738

**Published:** 2026-04-30

**Authors:** Richi Nakatake, Tetsuya Okuyama, Tominori Kimura, Mikio Nishizawa

**Affiliations:** 1 Department of Pancreatobiliary Surgery, Kansai Medical University, Hirakata, Osaka, Japan; 2 Comprehensive Research Organization for Science and Society, Ritsumeikan University, Kusatsu, Shiga, Japan; 3 College of Life Sciences, Ritsumeikan University, Kusatsu, Shiga, Japan; 4 Department of Biology, Faculty of Mathematics and Natural Sciences, Brawijaya University, Malang, Indonesia

**Keywords:** circular RNA, competing endogenous RNA, extracellular vesicle, methyladenosine, miRNA, natural antisense transcript, non-coding RNA, oligonucleotide

## Abstract

Natural antisense transcripts (NATs) from eukaryotic genes, known as long non-coding RNAs (lncRNAs), are long transcripts that do not encode proteins. NATs play diverse functional roles in regulating the transcription, stability, and translation of protein-coding genes at the epigenetic and post-transcriptional levels. Here, we outline recent studies on NAT-mediated RNA networks and discuss their potential as therapeutic targets across diseases. Interferon-α1 (*IFNA1*) mRNA expression is regulated by its overlapping antisense transcript *IFNA1-AS* through *IFNA1* mRNA–*AS* duplex formation, and microRNA-sponging through common microRNA response elements (MREs) as competing endogenous RNAs. The competitive interactions between NATs and mRNA MRE(s) fine-tune mRNA and protein levels. The receptor tyrosine kinase, ephrin type-A receptor 2 (*EPHA2*) mRNA and its antisense partner (*EPHA2-AS*) are transcribed from the *EPHA2* gene and are overexpressed in breast cancer. *EPHA2-AS* interacts with *EPHA2* mRNA, forming an mRNA–*AS* duplex that modulates both *EPHA2* mRNA and protein levels, potentially contributing to tumorigenesis; hence, it is a potential target for breast cancer treatment. RNA methylation, such as *N* ^6^-methyladenosine, may also play a role in regulating gene expression in various diseases. NAT-targeted therapeutics, such as synthetic oligonucleotides, mRNA, and drugs, can be introduced into cells either directly or via extracellular vesicles and lipid nanoparticles. The administration of NAT-targeted therapeutics in animal disease models is useful for evaluating their efficacy. The mechanisms of NAT-mediated gene regulation should be further investigated to develop NAT-targeted therapeutics for the treatment of various diseases.

## 1 Introduction

Natural antisense transcripts (NATs) transcribed from eukaryotic genes are primarily long (>200 nucleotides) transcripts that do not encode proteins, that is, long non-coding RNAs (lncRNAs). NATs are transcribed in the opposite direction to mRNA from the same gene and are distributed in the nucleus and cytoplasm. NATs are highly abundant in the human genome and are found at many gene loci (Krappinger et al., 2021; Seal et al., 2023).

Although NATs are heterogeneous and are mostly expressed at low levels, short-read sequencing (RNA sequencing) has enabled the identification of many lncRNAs, including NATs, as well as other regulatory RNAs.

**FIGURE 1:**
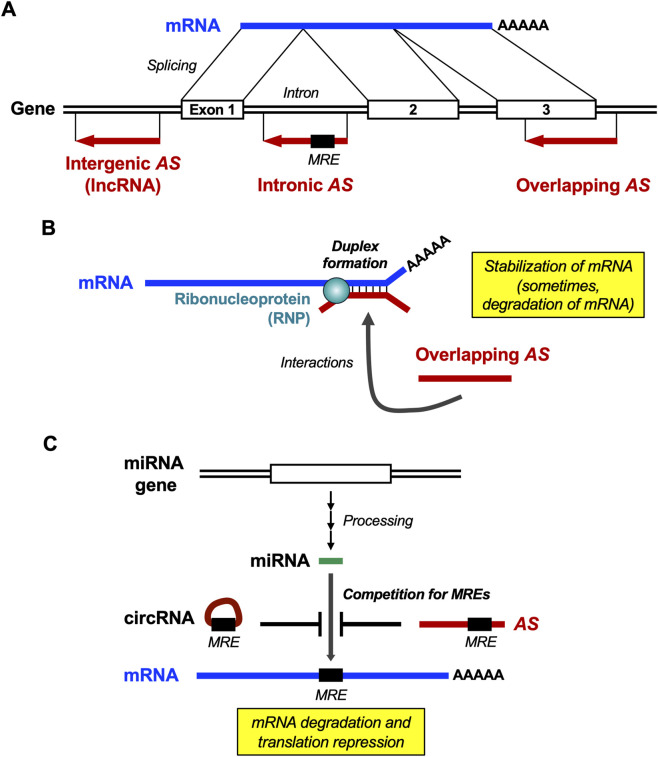
Messenger RNA (mRNA) and regulatory RNAs in cells. **(A)** Transcription of mRNA and NAT: An mRNA is transcribed from a gene, and then spliced, capped, and polyadenylated. NATs (shown as *AS*) synthesized from corresponding genes are >200 nucleotides in the length and do not code for proteins, that is, lncRNAs. Three types of NATs are intergenic, intronic, and overlapping NATs (Latgé et al., 2018; Krappinger et al., 2021). **(B)** RNA duplex formation between mRNA and NAT: An overlapping NAT hybridizes with an mRNA at a single-stranded region, such as a loop or bulge in the secondary structure to form a short mRNA–NAT duplex, which is then bound to RNP(s). Resultant complex stabilizes (sometimes destabilizes) the mRNA (Nishizawa et al., 2015; Nishizawa et al., 2022). **(C)** ceRNAs: miRNA is synthesized as a precursor from a given gene and then undergoes processing by Drosha and Dicer (Virtue et al., 2012). circRNA is also synthesized. MREs are located in mRNAs, NATs, and circRNA. Binding of miRNA to an MRE(s) of mRNA is competitively inhibited by NATs and circRNAs if they share the common MRE(s) (Karreth and Pandolfi, 2013; Tay et al., 2014; Kimura et al., 2015).

Based on their relative positions, NATs are classified into three types (Latgé et al., 2018; Krappinger et al., 2021): (1) *intergenic*, a NAT (lncRNA) located between two genes; (2) *intronic*, a NAT located in an intron; and (3) *overlapping*, a NAT overlapping with an mRNA and involving mutual interactions (Figure 1A). Standardized nomenclature was assigned to lncRNAs, including overlapping and intronic NATs (Seal et al., 2023). For example, the overlapping antisense transcript of the human interferon (*IFN*) gene is designated *IFN-AS* (Kimura et al., 2015).

NATs play diverse roles in regulating gene expression at the epigenetic, transcriptional, and post-transcriptional levels in the nucleus and cytoplasm (Wahlestedt, 2006; Nishizawa et al., 2015; Werner et al., 2024). NATs are regulatory RNA molecules implicated in epigenetic modifications, transcriptional interference, modulation of alternative splicing to mediate translational efficiency, mRNA stability, masking of microRNA response elements (MREs) to sponge microRNAs (miRNAs), and other mechanisms (Faghihi and Wahlestedt, 2009; Nishizawa et al., 2015; Krappinger et al., 2021). miRNAs modulate protein expression through mRNA degradation and translational repression (Virtue et al., 2012).

Two well-studied mechanisms of NAT actions are (1) an antisense transcript (overlapping NAT) and its relevant mRNA form an mRNA–NAT duplex at loops in the secondary structure, to which RNA-binding proteins (ribonucleoproteins, RNPs) bind to stabilize the mRNA (Matsui et al., 2008) (Figure 1B); and (2) common MRE(s) shared between antisense transcript and its mRNA. The NATs act as a competing endogenous (ce) RNA by sequestering MRE-shared miRNAs, which dowenregulate their expression (Karreth and Pandolfi, 2013; Tay et al., 2014) (Figure 1C). Consequently, NATs in the ceRNA network are involved in various pathological processes, including immune responses, neurodegenerative disorders, tumorigenesis and oncogenic progression (Krappinger et al., 2021).

Here, we focus on several examples of NAT-mediated mechanisms and provide an overview of the regulatory networks involving mRNAs, NATs, and other regulatory RNAs. We also discuss the biological functions and potential applications of NATs in the treatment of NAT-associated diseases.

## 2 NATs and their involvement in disease

### 2.1 Interferon-α1 (*IFNA1*) gene

Interferon-α1 is a type I interferon family member that is induced in response to viral infection as a key part of the innate immune response. The interferon-α1 (*IFNA1*) mRNA levels are post-transcriptionally regulated by its antisense transcript *IFNA1-AS*, which stabilizes *IFNA1* mRNA through cytoplasmic *IFNA1* mRNA–*AS* duplex formation at a single-stranded loop domain and enhances the accessibility of RNPs (Kimura et al., 2013) (Figure 1B). Another possible mechanism may involve this *IFNA1-AS*, as well as the mRNAs and antisense transcripts of other members of the *IFNA* multigene family, acting as ceRNAs via common MREs (Kimura et al., 2015) (Figure 1C). Therefore, *IFNA* mRNAs and *IFNA-AS* determine post-transcriptional *IFNA1* mRNA levels.

*IFNA-AS* represses the growth of human influenza A virus in a guinea pig model, harboring MX dynamin-like GTPase 1. Antisense oligoribonucleotides, which represent the functional domains of *IFNA1-AS,* inhibit influenza A virus proliferation in the respiratory tracts of virus-infected guinea pigs (Sakamoto et al., 2019). These antisense oligoribonucleotides mimicked *IFNA1-AS* and exhibited antiviral activity.

### 2.2 Ephrin type-A receptor 2 (*EPHA2*) gene

Several lncRNAs, including NATs, have been implicated in breast cancer development. Latgé et al. (2018) summarized these studies and discussed the molecular mechanisms underlying their functions. EPHA2 is a receptor tyrosine kinase that is overexpressed in breast and other cancers. *EPHA2* expression is thought to be causally related to tumorigenesis. Two splice variants of the NATs of *EPHA2* gene (*EPHA2-AS1/2*) overlap with *EPHA2* mRNA and may form *EPHA2* mRNA–*AS* duplexes. They modulate *EPHA2* mRNA levels in human breast adenocarcinoma cell lines and patient samples, with the highest levels detected in triple-negative breast cancer cells, such as MDA-MB-231 cells (Okuyama et al., 2020).

*EPHA2-AS1/2* modulates cytoplasmic *EPHA2* mRNA levels by interacting with a complementary single-stranded region of the mRNA specific to *AS1/2* in MDA-MB-231 cells. This result was confirmed by *EPHA2-AS1/2* silencing using a sense oligonucleotide or by overexpression of an antisense oligoribonucleotide, in which both the sense and antisense oligonucleotides were derived from the functional region of *EPHA2* mRNA targeted by *EPHA2-AS1/2* (Okuyama et al., 2020). These antisense transcripts promote the proliferation and migration of cells through EPHA2-dependent Ras signaling pathways. These findings suggest that *EPHA2-AS1/2* is a potential target for the triple-negative breast cancer treatment (Odaka et al., 2024).

### 2.3 Inducible nitric oxide synthase (*iNOS*) gene

Thus, NATs may play a crucial role in the pathophysiology of inflammatory diseases. iNOS (also known as NOS2) catalyzes the production of the proinflammatory mediator nitric oxide (NO). In sepsis, excessive NO production in hepatocytes and macrophages has been implicated in tissue injury (Nishizawa et al., 2015; Nishizawa et al., 2022). Similar to *EPHA2-AS1/2*, the overlapping antisense transcripts of *iNOS* gene (*iNOS-AS*) interact with and stabilize *iNOS* mRNA with RNPs (Matsui et al., 2008). When an oligonucleotide representing the loop of *iNOS* mRNA (i*NOS* sense oligonucleotide) targeted by *iNOS-AS* was introduced into rat hepatocytes, it decreased both *iNOS* mRNA and protein levels by interfering with mRNA–*AS* interactions in the cytoplasm (Nishizawa et al., 2015).

To target the mRNA–NAT duplex mechanism, NAT-targeted regulation technology (NATRE) using sense oligonucleotides may be applied in the treatment of animal disease models. A sepsis/endotoxemia model was established by administering D-galactosamine and bacterial lipopolysaccharide to rats. When an *iNOS* sense oligonucleotide was simultaneously administered with D-galactosamine and lipopolysaccharide to rats, the survival rate significantly increased. The *iNOS* and tumor necrosis factor-α mRNA levels were also decreased in the livers of the sense oligonucleotide-administered rats (Okuyama et al., 2018; Nishizawa et al., 2022).

Recombinant soluble thrombomodulin (rTM), an endothelial-type anticoagulation cofactor that inhibits intravascular coagulation by binding to thrombin, is used to treat disseminated intravascular coagulopathy (DIC) by suppressing coagulation, inflammation, and apoptosis. Because DIC, also known as sepsis-associated coagulopathy, is a frequent complication of sepsis, combination therapy with an *iNOS* sense oligonucleotide and rTM was evaluated for hepatoprotection in a rat model of septic shock after partial hepatectomy. The combination of the anti-DIC drug rTM and the *iNOS* sense oligonucleotide improved the survival of sepsis model rats compared with the sense oligonucleotide alone and reduced hepatic *iNOS* mRNA levels and serum NO concentrations; however, it remains unclear whether rTM affects *iNOS* mRNA*–AS* interactions (Nakatake et al., 2023). Collectively, sense oligonucleotides may act as nucleic acid drugs that suppress inflammatory responses, and their efficacy may be enhanced by other drugs with different mechanisms of action.

## 3 Other examples of NAT-mediated mechanisms

### 3.1 NATs and alternative splicing

Two typical NAT-mediated mechanisms that target mRNA–NAT duplexes and ceRNAs through MRE(s) are reviewed above. They function in the cytoplasm to regulate mRNA levels, whereas NATs also regulate gene expression in the nucleus. Other NAT-mediated mechanisms are discussed below.

NATs regulates the alternative splicing. The leukocyte common antigen *CD45* encodes protein tyrosine phosphatase receptor type C, which is expressed in all nucleated hematopoietic cells. The primary *CD45* transcript uses three alternative exons to produce four protein isoforms. The antisense transcript (*CD45-AS*, *PEBP1P3*) was transcribed in the opposite direction of the *CD45* gene (intron 2). Intronic *CD45-AS* regulates alternative splicing of *CD45* mRNA, possibly by regulating histone H3 modification (acetylation and methylation) and DNA methylation of *CD45* gene intron 2. *CD45-AS* also decreases the binding of the chromatin conformation organizer CCCTC-binding factor (CTCF) to intron 2 and alternative splicing exons of *CD45* gene. The expression levels of *CD45* splicing isoforms affect lymphocyte function and development and thus, immune system activity. Therefore, changes in the expression levels of *CD45* alternative splicing isoforms caused by *CD45-AS* may contribute to autoimmune diseases or immune deficiencies (Su et al., 2021).

### 3.2 Involvement of circular RNA (circRNA) in disease

CircRNAs are single-stranded, covalently closed RNA molecules that are ubiquitous across species ranging from viruses to mammals. CircRNAs classified as novel ncRNA species were identified by transcriptome analysis using RNA sequencing. Several circRNAs have been identified. Lariat RNAs, which are covalently closed and spliced from precursor mRNAs, are well-known intronic circRNAs. Due to their covalent ring structure and lack of free ends, circRNAs are highly stable ncRNAs in cells. circRNAs are expressed in mammalian cells in a tissue- or cell-specific manner and primarily function as miRNA sponges (Zhou et al., 2020). Because circRNAs compete with NATs when they share common MRE(s), circRNAs and NATs are ceRNAs.

circRNAs are involved in the development and progression of cancer, cardiovascular diseases, diabetes, and neurological disorders (Zhou et al., 2020; Karimi et al., 2025). NATs and their corresponding regulatory RNAs play crucial roles in the pathogenesis of neuropathic pain. Peripheral nerve injury or noxious stimuli can induce extensive changes in the expression of miRNAs, NATs, lncRNAs, and circRNAs. miRNAs regulate neuroinflammation during the progression of neuropathic pain. Antisense transcripts of early growth response 2 (*Egr2-AS*) and potassium voltage-gated channel subfamily A member 2 (*Kcna2-AS*) were upregulated in Schwann cells and dorsal root gangla, respectively. LncRNAs and NATs, such as X-inactive specific transcript (*XIST*) and nuclear paraspeckle assembly transcript 1 (*NEAT1*), are upregulated in the spinal cord, and *XIST* and *NEAT1* act as miRNA sponges. The circRNA circHIPK3 was abnormally expressed in the dorsal root ganglia, whereas ciRS-7, cirZNF609, and circ_0005075 were upregulated in the spinal cord. Sequestration of miRNAs by NATs and circRNAs leads to the expression of pain-related molecules or modulation of miRNA processing (Jiang et al., 2022).

### 3.3 NATs and RNA modification

Epigenetic modifications such as *N* ^6^-methyladenosine (m^6^A) and 5-methylcytosine (m^5^C) are frequently observed in various RNA transcripts. These modifications alter RNA structure and properties, thereby modulating lncRNA functions and interactions (Cusenza et al., 2023). The m^6^A modification of lncRNAs facilitates transcriptional activation (Lee et al., 2021). The binding of YTH domain-containing family protein 2 (YTHDF2), an m^6^A reader, to m^6^A leads to the degradation of m^6^A-containing mRNA (Wang et al., 2014). The functions of m^5^C are less clear, although its biological effects include regulation of RNA localization, stability, and transcription efficiency (Cusenza et al., 2023).

The m^6^A modification affects the transcription of many genes and has been implicated in cancer (Cusenza et al., 2023), diabetic nephropathy (Huang et al., 2024), and Alzheimer’s disease (Zhang et al., 2022). The antisense transcript of the *STEAP3* gene (*STEAP3-AS1*) is highly expressed in human colorectal cancer tissues. Zhou et al. (2022) reported that *STEAP3-AS1* is induced by hypoxia-inducible factor-1α (HIF-1α) and affects *STEAP3* mRNA stability by binding to the m^6^A reader YTHDF2. *STEAP3-AS1* competitively interacts with YTHDF2 to dissociate YTHDF2 from *STEAP3* mRNA and prevent m^6^A-mediated *STEAP3* mRNA degradation. High-level expression of the STEAP3 protein activates the Wnt/β-catenin signaling pathway and promotes colorectal cancer progression.

### 3.4 Involvement of NATs in extracellular vesicles (EVs) in cancer

EVs have been studied extensively over the past decade. EVs, including exosomes (endosome-derived particles with plasma membranes, classified as small EV), are secreted by cells into the extracellular space, i.e., their microenvironment and systemic circulation such as blood and urine (Jeppesen et al., 2023). EVs carry cell-derived proteins and RNAs (mRNAs, NATs, and miRNAs) and transport them to other cells (Miceli et al., 2024). The EV cargo detected by liquid biopsy may seerve as disease biomarkers. EV uptake generally occurs in the liver, kidneys, spleen, lungs, colon, and bones (Jeppesen et al., 2023). EVs are involved in intercellular communication with other cells. The incorporation of NATs from cancer cell-derived EVs into other (acceptor) cells may promote cell growth and metastasis.

Abnormal NAT expression of the actin filament-associated protein 1 gene (*AFAP1-AS*) and lncRNAs, including *H19* and metastasis-associated lung adenocarcinoma transcript 1 (*MALAT1*), is observed in endometriosis, a common gynecological disease. Additionally, the antisense transcript of *HIF1A* gene (*HIF-AS*, *aHIF*) is highly expressed in the serum EVs of patients with endometriosis. These NATs can be transported through small EVs and subsequently introduced into other cells. These transferred endometriosis-related NATs can sponge miRNAs, promoting the invasion and metastasis of endometrial stromal cells and leading to endometriosis progression (Liu et al., 2023).

Solute carrier family 16 member 1 (SLC16A1) accelerates lactate influx and induces M2 macrophage polarization. Small EVs from hepatocellular carcinoma (HCC) cells carry NAT (*SLC16A1-AS1*), which stabilizes *SLC16A1* mRNA by facilitating its interaction with heterogeneous nuclear ribonucleoprotein A1 (HNRNPA1). IL-6 secreted by M2 macrophages activates signal transducer and activator of transcription 3 (STAT3) to induce methyltransferase 3 (*METTL3*) transcription in HCC cells. Increased METTL3 enhances m^6^A methylation of *SLC16A1-AS1*, and m^6^A-methylated *SLC16A1-AS1* increases the stability of *SLC16A1* mRNA. When *SLC16A1-AS1* in EVs is incorporated into other HCC cells, it promotes progression toward malignancy (Hu et al., 2024).

Finally, nodal growth differentiation factor (NODAL) is a transforming growth factor-β superfamily member and required during early embryonic development. NODAL is also involved in tumor progression and metastasis. *NODAL-AS* (*LADON*), a NAT that overlaps with exon 2 of *NODAL* gene, is highly expressed in metastatic melanoma cell lines. *NODAL-AS* may interact with *NODAL* mRNA, resulting in the upregulation of oncogenes and the downregulation of metastasis suppressor genes. *NODAL-AS* is enriched in small EVs derived from melanoma cells; *NODAL-AS* are incorporated to other melanoma cells, promoting tumor progression and metastasis. Collectively, *NODAL-AS* regulates melanoma progression (Dutriaux et al., 2023).

## 4 Discussion

### 4.1 RNA therapeutics based on mRNA–NAT duplex formation hypothesis

Several reports have demonstrated the therapeutic potential of targeting mRNA–NAT duplex formation (Figure 1B). Oligonucleotides that hybridize with mRNA and/or NAT are good drug candidates. As hybridization is highly specific to genes, these oligonucleotides (including DNA, RNA, and synthetic nucleic acids) may be used as drugs to treat diseases. Nucleic acid-based therapeutics belong to the third major drug class after low-molecular-weight (LMW) drugs and antibody-based therapeutics; therefore, their specificity and efficacy should be examined and compared.

Nucleic acid-based therapeutics are expected to affect complex RNA–protein networks among mRNA and regulatory RNAs, including NATs. Several difficulties must be overcome to develop new drugs, including optimizing their nucleotide sequences to avoid off-target effects, chemically modifying them to increase nuclease resistance, and incorporating them into cells. These points have been discussed in detail using *iNOS* sense oligonucleotides (Yoshigai et al., 2013).

RNPs such as HuR and hnRNPs contribute to the interactions among mRNAs, NATs, circRNAs, and miRNAs. NATs and other RNAs regulate mRNA levels in concert with RNPs. Therefore, the involvement of RNPs should be considered when investigating the NAT-mediated mechanisms.

Another approach involves controlling the NAT-mediated network by targeting mRNA–NAT duplexes using LMW molecules. For example, dexamethasone, a synthetic glucocorticoid, destabilizes *iNOS* mRNA, possibly by modulating the interactions among mRNA, NAT, and RNP (Ozaki et al., 2010). Pharmacologically active LMW compounds underlie the effects of traditional Japanese (Kampo) medicines and functional foods. Similar to sense oligonucleotides, LMW compounds may also recognize RNA structures (stem-loop structures and mRNA–*AS* duplexes) that affect mRNA stability (Nishizawa et al., 2022). AHCC® (a standardized extract of cultured *Lentinula edodes* mycelia) downregulated both *EPHA2-AS1/2* and *EPHA2* mRNA, possibly by reducing *EPHA2* mRNA–*AS* duplex (Odaka et al., 2024).

### 4.2 RNA therapeutics based on ceRNA hypothesis

The ceRNA mechanism is another potential target for disease treatment. *IFNA1-AS*, mRNAs, and NATs of the other *IFNA* multigene family members serve as ceRNAs (Kimura et al., 2015). Pseudogenes, lncRNAs, and circRNAs also serve as ceRNAs. Crosstalk between ceRNAs and mRNAs via shared MREs plays an important role in the pathophysiology of various diseases. RNA pharmaceuticals based on pain-related NATs and circRNAs may serve as novel analgesics for treating neuropathic pain by sequestering miRNAs.

EVs can be used to deliver nucleic acid-based drugs. As endometriosis-related NATs promote the invasion and metastasis of endometrial stromal cells by sponging miRNAs (Liu et al., 2023), endometriosis progression may be suppressed by lowering NATs. Thus, cancer-related NATs may be suitable targets for cancer treatment.

Recent RNA sequencing data have indicated the presence of many NATs and circRNAs in cells (Zhou et al., 2020). These findings raise complex questions: how do NATs interact with circRNAs, and why are so many circRNAs involved in gene regulation and disease pathophysiology? However, the functions and biological roles of NATs and circRNAs require further investigation.

### 4.3 Applications of RNA therapeutics to human diseases

Cell-based studies cannot address problems related to *in vivo* efficacy, off-target effects, and organ delivery. Therefore, animal experiments are essential for evaluating nucleic acid-based drugs. Rat models of sepsis/endotoxemia can be used to assess the effects of nucleic acid-based therapeutics; for example to evaluate an *iNOS* sense oligonucleotide targeting *iNOS-AS* (Okuyama et al., 2018).

*IFNA1* antisense oligoribonucleotides exhibited antiviral effects in influenza A virus-infected guinea pigs (Sakamoto et al., 2019). When the functional domains of a NAT are known, as in the case of *IFNA1-AS*, antisense oligoribonucleotides act as NATs and manifest their effects. Because dysfunction of *IFNA1-AS* may contribute to autoimmunity by failing to sustain *IFNA1* gene expression and function, targeting *IFNA1-AS* could modulate overstimulated innate immune pathways to treat inflammatory autoimmune disorders, such as autoimmune cutaneous diseases (Muntyanu et al., 2022).

**FIGURE 2:**
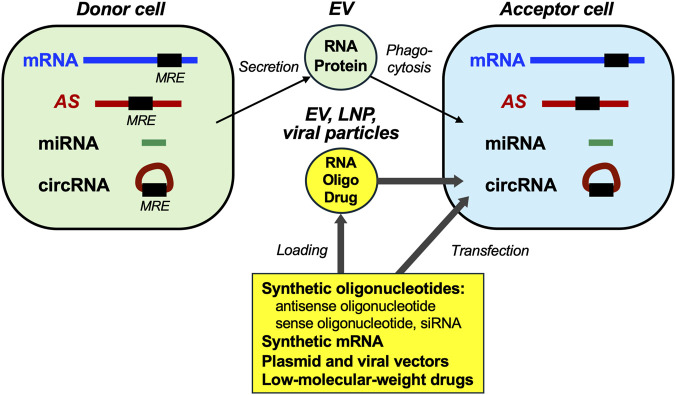
Intercellular communication mediated by extracellular vesicles and introduction of nucleic acids and drugs. In the cells, mRNA, NATs (*AS*), miRNA, and circRNA are present and mutually interacted. MRE(s) may exist in mRNA and regulatory RNAs. EVs, which include RNA and protein are secreted from the donor cell and transported to the acceptor cell. When the donor cell is a cancer cell, features of the acceptor cell may be affected by the EV transport. RNA therapeutics (synthetic oligonucleotides, mRNA, and LMW drugs, as well as plasmids and viral vectors) can be directly introduced to the acceptor cell using transfection or infection. Alternatively, they can be loaded onto EVs or LNPs and are transported to the acceptor cell, such as cancer cells. Thin arrows represent endogenous transport pathways of EVs; thick arrows represent introduction of exogenous molecules using EVs, LNPs, or viral particles to alter endogenous mRNA levels in the acceptor cells. These nanotechnologies may be applied to the administration of RNA therapeutics to humans.

Khorkova et al. (2022) reviewed commercially available nucleic acid-based therapeutics to humans, including antisense oligonucleotides and short-interfering RNAs. Nucleic acid-based and/or LMW drugs can be loaded onto EVs and lipid nanoparticles (LNPs) to target specific genes and control their expression. EVs and LNPs are potent carriers of synthetic oligonucleotides and mRNAs in cells (Jeppesen et al., 2023) (Figure 2). These nanotechnologies offer robust platforms for precison-drug delivery to various cancers, including HCC (Shi et al., 2026).

Rapidly emerging findings on the intercellular communication of NATs should also be considered. NATs and miRNAs are transported in EVs and alter signal transduction and gene expression in the acceptor cell, leading to malignant effects such as promotion of cell growth and metastasis. The application of LNP-encapsulated small-interfering Ythdf1 significantly improves the efficacy of anti-PD-1 therapy in metabolic dysfunction-associated steatohepatitis (MASH)-HCC allograft models (Wang et al., 2023).

Overall, NAT-based regulatory networks play crucial roles in immune response, inflammation, and tumorigenesis. Various approaches are essential to better understand NAT-mediated networks and to develop nucleic acid-based drugs for disease.
